# Brain-machine interface-based training for improving upper extremity function after stroke: A meta-analysis of randomized controlled trials

**DOI:** 10.3389/fnins.2022.949575

**Published:** 2022-08-03

**Authors:** Yu-lei Xie, Yu-xuan Yang, Hong Jiang, Xing-Yu Duan, Li-jing Gu, Wu Qing, Bo Zhang, Yin-xu Wang

**Affiliations:** ^1^Department of Rehabilitation Medicine, Affiliated Hospital of North Sichuan Medical College, Nanchong, China; ^2^Department of Rehabilitation Medicine, Capital Medical University, Beijing, China; ^3^Department of Rehabilitation Medicine, The Second Clinical Hospital of North Sichuan Medical College, Nanchong Central Hospital, Nanchong, China; ^4^Department of Rehabilitation Medicine, Xichong County People's Hospital, Nanchong Central Hospital, Nanchong, China

**Keywords:** upper limb dysfunction, stroke, meta-analysis, systematic review, brain-machine interface

## Abstract

**Background:**

Upper extremity dysfunction after stroke is an urgent clinical problem that greatly affects patients' daily life and reduces their quality of life. As an emerging rehabilitation method, brain-machine interface (BMI)-based training can extract brain signals and provide feedback to form a closed-loop rehabilitation, which is currently being studied for functional restoration after stroke. However, there is no reliable medical evidence to support the effect of BMI-based training on upper extremity function after stroke. This review aimed to evaluate the efficacy and safety of BMI-based training for improving upper extremity function after stroke, as well as potential differences in efficacy of different external devices.

**Methods:**

English-language literature published before April 1, 2022, was searched in five electronic databases using search terms including “brain-computer/machine interface”, “stroke” and “upper extremity.” The identified articles were screened, data were extracted, and the methodological quality of the included trials was assessed. Meta-analysis was performed using RevMan 5.4.1 software. The GRADE method was used to assess the quality of the evidence.

**Results:**

A total of 17 studies with 410 post-stroke patients were included. Meta-analysis showed that BMI-based training significantly improved upper extremity motor function [standardized mean difference (SMD) = 0.62; 95% confidence interval (CI) (0.34, 0.90); *I*^2^ = 38%; *p* < 0.0001; *n* = 385; random-effects model; moderate-quality evidence]. Subgroup meta-analysis indicated that BMI-based training significantly improves upper extremity motor function in both chronic [SMD = 0.68; 95% CI (0.32, 1.03), *I*^2^ = 46%; *p* = 0.0002, random-effects model] and subacute [SMD = 1.11; 95%CI (0.22, 1.99); *I*^2^ = 76%; *p* = 0.01; random-effects model] stroke patients compared with control interventions, and using functional electrical stimulation (FES) [SMD = 1.11; 95% CI (0.67, 1.54); *I*^2^ = 11%; *p* < 0.00001; random-effects model]or visual feedback [SMD = 0.66; 95% CI (0.2, 1.12); *I*^2^ = 4%; *p* = 0.005; random-effects model;] as the feedback devices in BMI training was more effective than using robot. In addition, BMI-based training was more effective in improving patients' activities of daily living (ADL) than control interventions [SMD = 1.12; 95% CI (0.65, 1.60); *I*^2^ = 0%; *p* < 0.00001; *n* = 80; random-effects model]. There was no statistical difference in the dropout rate and adverse effects between the BMI-based training group and the control group.

**Conclusion:**

BMI-based training improved upper limb motor function and ADL in post-stroke patients. BMI combined with FES or visual feedback may be a better combination for functional recovery than robot. BMI-based trainings are well-tolerated and associated with mild adverse effects.

## Introduction

Stroke is the second cause of death and the leading cause of disability worldwide (GBD Stroke Collaborators, 2021). Most stroke survivors suffer varying degrees of disability, which can greatly affect their functional independence, quality of life, and increase the burden of nursing care (Bejot et al., 2016; Mutai et al., 2016; Purton et al., 2021). Of these, nearly 80% of post-stroke persons have upper extremity dysfunction that gravely affects their ability to perform activities of daily living (ADL), as well as social participation (Feigin, 2008). Various rehabilitation approaches have been proposed to promote upper limb motor recovery, such as constraint-induced motor therapy (CIMT), and task-oriented training (Winstein et al., 2016). However, these rehabilitation strategies are limited for individuals with severe motor dysfunction (Corbetta et al., 2015). For such patients, mirror therapy (Thieme et al., 2018), motor imagery (Guerra et al., 2017), action observation therapy (Ertelt et al., 2007; Tani et al., 2018), electrical/magnetic stimulation (Dawson et al., 2016; Kang et al., 2016; Lu et al., 2022) (e.g., non-invasive brain stimulation, or vagus nerve stimulation) are alternative approaches and those may enhance the reorganization of function in damaged neural networks to minimize motor deficits. In recent years, novel rehabilitation technologies, such as virtual reality (Ikbali Afsar et al., 2018), robot-assisted therapy, and BMI-based training, have been proposed for post-stroke rehabilitation, which can active engagement of patients as well as their motivation compared to conventional rehabilitation (Levin et al., 2010).

BMI is a novel technique that captures brain activity while performing or attempting to perform motor and/or cognitive tasks, and processing it into useful feedback (Chamola et al., 2020). This feedback can be expressed in many forms, including feedback of a visual (Verbaarschot et al., 2021), auditory (Hubner et al., 2018) or tactile nature (Fleury et al., 2020), or even control signals for external devices (Vilela and Hochberg, 2020). BMI can be invasive or non-invasive and due to safety and ethical issues, non-invasive may be more promising for clinical applications. Among non-invasive systems, EEG is one of the best candidates because of its low cost, portability, and ability to extract many signal features that convey brain activity (van Dokkum et al., 2015). Sensorimotor rhythm (SMR), an oscillatory rhythm of synchronized brain activity of 8–30 Hz above the sensorimotor cortex that change with movement and/or movement imagery (Lu et al., 2013), is a critical EEG signal that correlates closely with motor area activation during real and imagined movements (McFarland and Wolpaw, 2011). Pattern changes in SMR amplitude (asynchronous or synchronous) can trigger external devices through the BMI system to display real-time sensory feedback or perform predetermined actions. In general, BMI can be divided into two categories according to its role: being an effective tool to replace or substitute for a lost function (Assistive BMI), such as assistive communication tools for people with paralysis (Chaudhary et al., 2021), and serving as a novel technology to strengthen damaged neural pathways or induce cortical plasticity (Rehabilitative BMI).

In post-stroke patients, we prefer to use BMI in training to promote neuroplasticity and motor regeneration. Since the first case report on the feasibility of combining BMI and FES for post-stroke patients in 2009 (Daly et al., 2009), a growing number of human BMI studies demonstrate the great potential of this technology in restoring movement (Lyukmanov et al., 2018; Chung et al., 2020; Li et al., 2021). Most post-stroke upper limb rehabilitation programs use non-invasive BMI systems combined with feedback devices, such as FES (Li et al., 2014; Kim et al., 2016; Biasiucci et al., 2018; Lee et al., 2020), robot (Ang et al., 2015; Cheng et al., 2020), or visual display (Mihara et al., 2013; Pichiorri et al., 2015) to facilitate motor recovery. However, the controversy surrounding the effectiveness of BMI-based training for post-stroke upper extremity dysfunction persists due to the different types of experimental clinical studies and the wide variation in the quality of the literature. In recent years, although some meta-analyses have investigated the effect of BMI-based training on upper limb motor function after stroke (Cervera et al., 2018; Bai et al., 2020; Yang W. et al., 2021; Mansour et al., 2022; Nojima et al., 2022), there are limitations such as the small sample size included in the study and the failure to provide a level of evidence. In addition, we found data extraction errors in a study analyzing the efficacy of BMI in different stroke stages (Yang W. et al., 2021). The evidence related to the immediate and/or long-term effects of BMI in other aspects (e.g., spasticity, activities of daily living, etc.) is also inconclusive.

In this review, we performed a more comprehensive inclusion (17RCTs) covering RCTs from previously published meta-analyses, in addition to two uncited studies Wang et al. (2018) and Li et al. (2022). We aim to provide updated and higher quality evidence on the effectiveness and safety of BMI-based training in improving function in post-stroke patients. We focus the meta-analysis on the following aspects: (1) to investigate the immediate clinical effects of BMI-based training on improving upper limb motor function in hemiplegia; (2) to investigate the potential differences in treatment effects caused by different external device of BMI (e.g., BMI combined with FES and BMI combined with robot); (3) to investigate the immediate clinical effects of BMI-based training on improving activities of daily living in post-stroke patients. In addition, we analyzed adverse effects and shedding in RCTs to provide evidence for the safety and acceptance of this novel technology, considering its widespread use in clinical settings.

## Materials and methods

The review process was confirmed using a checklist in the PRISMA statement for reviews (Ardern et al., 2022).

### Search strategies

The meta-analysis included RCTs that investigated the effects of BMI-based training on upper limb function after stroke which were published in peer-reviewed journals and in English. Articles were retrieved from five databases until April 1, 2022 (PubMed, Web of Science, Embase, Scopus, and Cochrane Library) using the following keywords that included “upper extremity,” “stroke,” and “brain-computer/machine interface.” We also performed a manual search, including screening reference lists of previous systematic reviews and meta-analysis, to retrieve additional relevant articles for analysis.

### Inclusion and exclusion criteria

Two review authors independently assessed the methodological quality of the included studies. We recorded and resolved any disagreements through discussions with a third reviewer.

The inclusion criteria for this study were as follows:

(1) Subjects of the study were adults with stroke diagnosed by computed tomography (CT) or magnetic resonance imaging (MRI).
(2) A randomized controlled trial.
(3) BMI training was performed in more than one session.
(4) the experimental group involving BMI intervention for the upper limb and the control intervention could be sham BMI training or conventional training without BMI
(5) used Fugl–Meyer Assessment Scale (FMA) or Modified Brathel Index (MBI) as an outcome measure.

The exclusion criteria for this study were as follows:

(1) Studies in which both experimental and control groups used BMI therapy.
(2) Neither pre-and post-intervention FMA nor MBI scores were available.
(3) redundant report.

### Data extraction

Duplicates were first eliminated and the remaining retrieved articles were independently screened by two researchers using the same inclusion and exclusion criteria by reviewing titles and abstracts, and eligible articles were screened in full for final inclusion. Articles for which the abstract did not provide sufficient information were selected for full-text analysis. Discrepancies were resolved through discussion or consultation with a third investigator.

The following information was extracted from each study. (1) publication information (study authors, year of publication); (2) participant characteristics (age, duration of onset, and sample size) (3) intervention information (type of BMI-based intervention, intervention in the control group, and intervention parameters); and (4) outcome measures [Fugl–Meyer Assessment Scale of Upper Extremity (FMA-UE), MBI, adverse effects and dropout rate].

Due to differences in the duration of intervention and follow-up assessments among studies, we extracted the pre-and post-intervention assessments as parameters for analysis of immediate clinical effects. Similarly, only adverse effects and shedding that occurred during the intervention were counted.

### Risk of bias and quality of outcomes assessment

Two investigators independently reviewed the included studies and assessed the methodological quality, a third reviewer recorded and resolved any discrepancies in the results. Because all the studies were RCTs, the Cochrane Handbook for Systematic Reviews of Interventions was used to assess the risk of bias, consisting of random sequence generation, allocation concealment, blinding of participants and personnel, blinding of outcome assessment, incomplete outcome data, selective reporting, and other sources of bias (Higgins et al., 2011; Corbett et al., 2014). The Grading of Recommendations Assessment, Development and Evaluation (GRADE) guidelines for systematic reviews were used to evaluate the quality of outcomes (Guyatt et al., 2008).

### Outcome measures

Outcome measures for the efficacy of therapy were as follows: (1) FMA-UE; (2) MBI; (3) adverse effects; (4) dropout rate.

The FMA-UE, with a sum of 66 points, is a well-designed, valid, and feasible assessment scale that is now widely used in the clinical assessment of upper extremity motor function (Gladstone et al., 2002; Platz et al., 2005). MBI is a five-level rating scale and evaluates the functional independence and autonomy of the subjects in 10 activities, including (1) bathing (2) personal grooming (3) feeding (4) dressing and undressing (5) bowel, and (6) bladder continence (7) getting on/off the toilet (8) stair climbing (9) moving from wheelchair to bed and return (10) walking. With high reliability and stability in people of different sexes and ages (Yang H. et al., 2021).

### Statistical analyses

All statistical analysis used the RevMan 5.4.1 statistical software (The Nordic Cochrane Center, The Cochrane Collaboration, Copenhagen, Denmark). For the continuous variable, FMA-UE and MBI were expressed as standardized mean differences (SMD) and 95% confidence intervals (CIs). If the mean change score and standardized differences (SD) were not available, but the assessment results regarding pre-and post-intervention were available, we transformed the pre/post-intervention scores to a mean change score and SD following the recommendation in the Cochrane Handbook for Systematic Reviews of Interventions. For dichotomous variables (adverse events and shedding), risk ratios (RR) or odds ratio (OR) and 95% CIs were used as statistical tools for efficacy analysis and effect sizes, respectively. The effect size was quantified as large (SMD > 0.8), medium (SMD 0.5–0.8), or small (SMD 0.2–0.5).

Heterogeneity in the intervention effect was inevitable because of the different study designs and the *I*^2^ statistic was used as a measure of heterogeneity indicating the percentage of total variability in a set of effect sizes caused by true heterogeneity (i.e., variation between studies) (Huedo-Medina et al., 2006). Values of *I*^2^ were used at 25, 50, and 75% to represent low, moderate, and considerable heterogeneity, respectively. A fixed-effects model for data pooling was used if the *I*^2^ statistic was below 50%, which meant that there was acceptable heterogeneity across the included studies. In contrast, the random-effects model was used if the *I*^2^ statistic was above 50% and then subgroup analysis or sensitivity analysis was performed to determine the source of heterogeneity.

## Results

### Literature search and study characteristics

**Figure 1:**
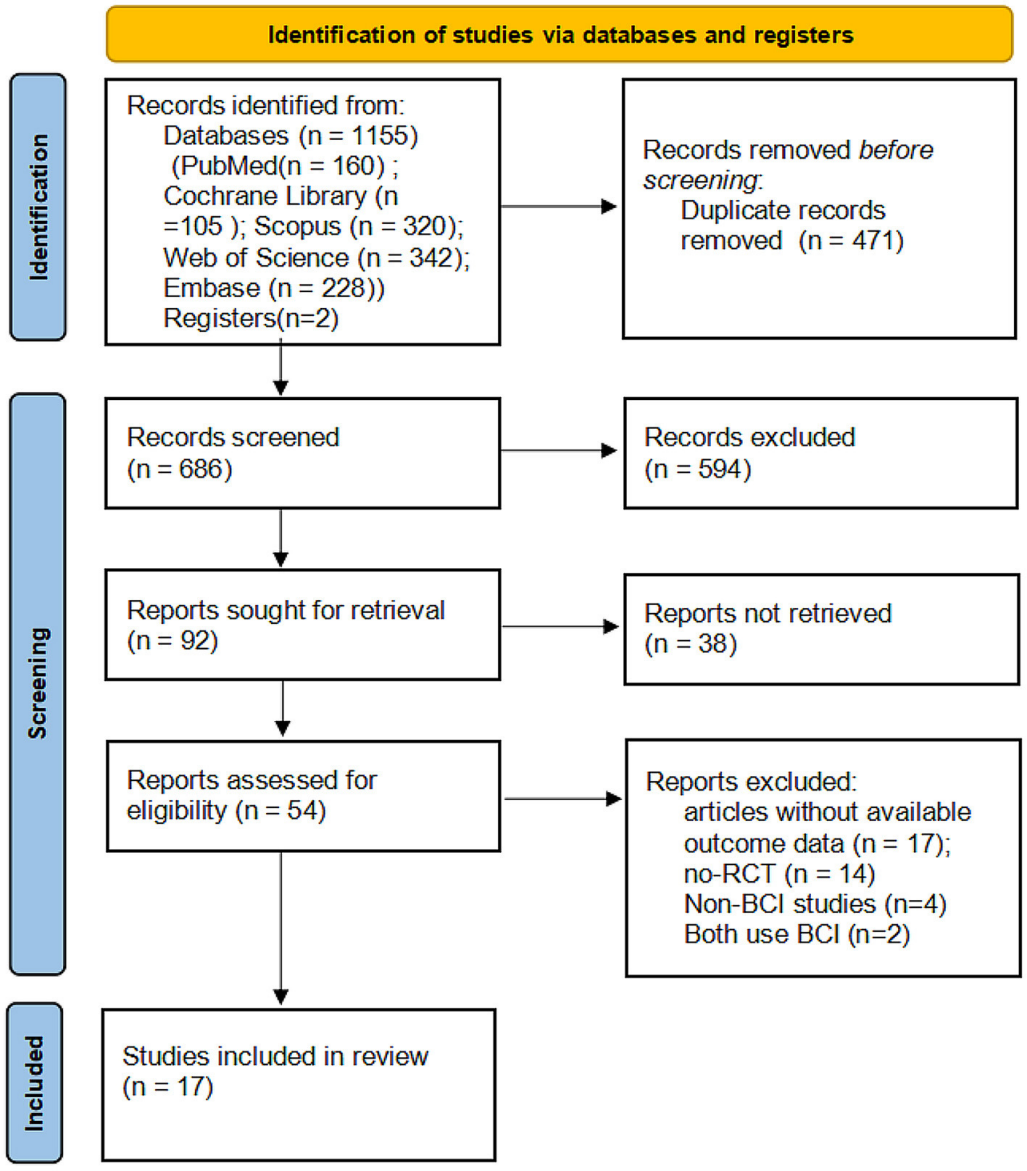
PRISMA flowchart of the study selection process. RCT, randomized controlled trials; BMI, brain-machine interface.

A flowchart depicting the selected studies is shown in Figure 1. We obtained 1,155 articles from the database search and additional records, and after adjusting for duplicated articles, the titles and abstracts of 594 publications were screened. Of these, 54 articles were assessed for eligibility by full-text screening, and 37 articles were excluded based on the inclusion criteria. The remaining 17 articles were retained for qualitative synthesis.

Table 1 shows the characteristics of the 17 studies included in this meta-analysis with a total of 410 participants (study sample sizes ranging from 10 to 74). Subjects included in this review were patients with first ischemic or hemorrhagic stroke (cortical and subcortical) confirmed by CT or MRI with a moderate-to-severe disability. The mean age ranged from 41.6 ± 12.0 to 66.3 ± 4.53. With 6 months as the cut-off point between the subacute and chronic phases of the stroke, 11 studies were conducted on chronic stroke patients (Mihara et al., 2013; Ramos-Murguialday et al., 2013; Ang et al., 2014, 2015; Kim et al., 2016; Biasiucci et al., 2018; Lin et al., 2018; Wang et al., 2018; Cheng et al., 2020; Lee et al., 2020; Miao et al., 2020), four studies were conducted on subacute stroke patients (Li et al., 2014; Pichiorri et al., 2015; Wu et al., 2019; Chen et al., 2020), and 2 studies included both chronic and subacute stroke patients (Frolov et al., 2017; Li et al., 2022). In 16 of the 17 studies, the BMI system relied on electroencephalography (EEG) to detect task-related changes in SMR. Another study used near-infrared spectroscopy (NIRS) to measure task-related changes in oxygen production and deoxygenated hemoglobin levels from the sensorimotor cortex (Mihara et al., 2013). Signals of detected motor intention were used to trigger sensory feedback provided by external devices [orthotics, robotics, functional electrical stimulation, tactile knobs, auditory, visual feedback, or Motion Tracking Device-Virtual Reality (MTD-VR)]. The duration of treatment ranged from 2 to 8 weeks, with most studies intervening for 4 weeks and total BMI-based treatment time ranging from 2 to 27 h.

**Table 1:** Characteristics of the randomized controlled studies.

**References**	***N***		**Age [mean** ±**sd or mean (25%, 75% quartiles)]**		**Time from stroke**		**Type of interventions**		**Intervention time (total session h)**	**Outcome Measures**
	**T**	**C**	**T**	**C**	**T**	**C**	**T**	**C**		
Ang et al. (2014)	6	7	54.0 ± 8.9	58.0 ± 19.3	285.7 ± 64.0(d)	455.4 ± 109.6(d)	MI-BCI (EEG) -Haptic Knob	Std Arm Th	1.5 h/d, 3 d/wk, 6 wk (27 h)	FMA-UE
Ang et al. (2015)	11	14	48.5 ± 13.5	53.6 ± 9.5	383.0 ± 290.8(d)	234.7 ± 183.8(d)	MI-BCI (EEG)-Robotic Feedback	Robot	1.5 h/d, 3 d/wk, 4 wk (18 h)	FMA-UE
Biasiucci et al. (2018)	14	13	56.4 ± 9.9	59.0 ± 12.4	39.8 ± 45.9(m)	33.5 ± 30.5(m)	BCI(EEG)-FES	Sham-FES	1 h/d, 2 d/wk, 5 wk (10 h)	FMA-UE, MRC, MAS, ESS
Chen et al. (2020)	7	7	41.6 ± 12.0	52.0 ± 11.1	3.1± 1.7(m)	3.9 ± 1.5(m)	MI-BCI (EEG)-Exoskeleton	MI	3 d/wk, 4 wk	FMA-UE
Cheng et al. (2020)	5	5	62.4 ± 4.7	61.4 ± 4.5	476.8 ± 302.0(d)	890.2 ± 257.23(d)	MI-BCI(EEG)—Soft Robotic	Soft robotic	1.5 h/d, 3 d/wk, 6 wk (27 h)	FMA-UE, ARAT
Frolov et al. (2017)	55	19	58.0 (48.0; 65.0)	58.0 (52.0; 67.0)	8.0 (4.0; 13.0)(m)	8.0 (1.0; 13.0)(m)	MI-BCI (EEG)-Exoskeleton	Sham- BCI-Exoskeleton	0.5 h/d, 5 d/wk, 2 wk (5 h)	ARAT, FMMA
Kim et al. (2016)	15	15	59.1 ± 8.1	59.9 ± 9.8	8.27 ± 1.98(m)	7.80 ± 1.78(m)	AOT-BCI(EEG)-FES	Conventional Treatment	0.5 h/d, 5 d/wk, 4 wk (10 h)	FMA-UA, MAL, ROM, MBI
Lee et al. (2020)	13	13	55.15 ± 11.57	58.30 ± 9.19	7.46 ± 1.61(m)	8.30 ± 1.97(m)	AOT-BCI(EEG)-FES	FES	0.5 h/d, 5 d/wk, 4 wk (10 h)	FMA-UE, WMFT, MBI, MAL
Li et al. (2014)	7	7	66.3 ± 4.53	67.1 ± 5.51	2.21 ± 1.69(m)	2.79 ± 1.85(m)	MI-BCI (EEG)-FES	FES	1 h/d, 3 d/wk, 8 wk (24 h)	FMA-UE, ARAT,
Li et al. (2022)	12	12	43.8 ± 14.7	55.0 ± 12.2	4.0 (2.0, 11.3)(m)	4.3 ± 2.6(m)	MI-BCI (EEG)-Robotic, Auditory, Visual Feedback	Conventional Treatment	1 h/d, 5 d/wk, 2 wk (5 h)	FMA-UE, WMFT, MBI
Lin et al. (2018)	5	5	45.0 ± 11.2	52.2 ± 7.7	17.8 ± 15.3(m)	10.8 ± 5.1(m)	BCI(EEG)- MTD-VR	Conventional Treatment	35 min/d, 3 d/wk, 4 wk (7 h)	FMA-UE
Miao et al. (2020)	8	8	48.8 ± 16.7	50.3 ± 17.1	18.3 ± 10.9(m)	11.1 ± 5.0(m)	MI-BCI (EEG)-FES	Conventional Treatment	3 d/wk, 4 wk	FMA-UE
Mihara et al. (2013)	10	10	56.1 ± 7.9	60.1 ± 8.5	146.6 ± 36.2(d)	123.4 ± 38.3(d)	MI-BCI (NIRS) -Visual Feedback	Sham- BCI	20 min/d, 3 d/wk, 2 wk (2 h)	FMA-UE, ARAT, MAL, KVIQ-10
Pichiorri et al. (2015)	14	14	64.1 ± 8.4	59.6 ± 12.7	2.7 ± 1.7(m)	2.5 ± 1.2(m)	MI-BCI (EEG) -Visual Feedback	MI	30 min/d, 3 d/wk, 4 wk (6 h)	FMA-UE, MAS, MRC, NIHSS
Ramos-Murguialday et al. (2013)	16	14	49.3 ± 12.5	50.3 ± 12.2	66 ± 45(m)	71 ± 72(m)	MI-BCI (EEG) -Orthosis	Sham- BCI	40 min/d, 5 d/wk, 4 wk (13.4 h)	FMA-UE, MAS, GAS
Wu et al. (2019)	14	11	62.93 ± 10.56	64.82 ± 7.22	2.11 ± 0.30(m)	2.00 (1.50, 3.00)(m)	MI-BCI (EEG)-Exoskeleton	Conventional Treatment	1 h/d, 5 d/wk, 4 wk (20 h)	FMA-UE, ARAT, WMFT
Wang et al. (2018)	13	11	54 ± 9	54 ± 9	3.73 ± 3.81(y)	3.55 ± 2.02(y)	AOT-BCI (EEG)-Robot	Robot	3–5 sessions/wk, 5–7 wk, 20 sessions	FMA-UE

T, experimental group; C, control group; min, minutes; d, days; wk, weeks; m, months; y, years; MI, Motor Imagery; AOT, Action Observational Training; EEG, Electroencephalography; NIRS, Near-Infrared Spectroscopy; BCI, Brain Computer Interface; MTD-VR, Motion Tracking Device-Virtual Reality; FES, Functional Electrical Stimulation; Std Arm Th, Standard Arm Therapy; FMA-UE, Fugl–Meyer Assessment Scale of Upper Extremity; MRC, Medical Research Council Scale; MAS, Modified Ashworth Scale; MBI, Modified Barthel Index; ROM, range of motion; GAS, Goal Attainment Scale; ARAT, Action Research Arm Test; WMFT, Wolf Motor Function Test. NIHSS, National Institute of Health Stroke Scale; MAL, Motor Activity Log; KVIQ-10, Kinesthetic and Visual Imagery Questionnaire.

**Figure 2:**
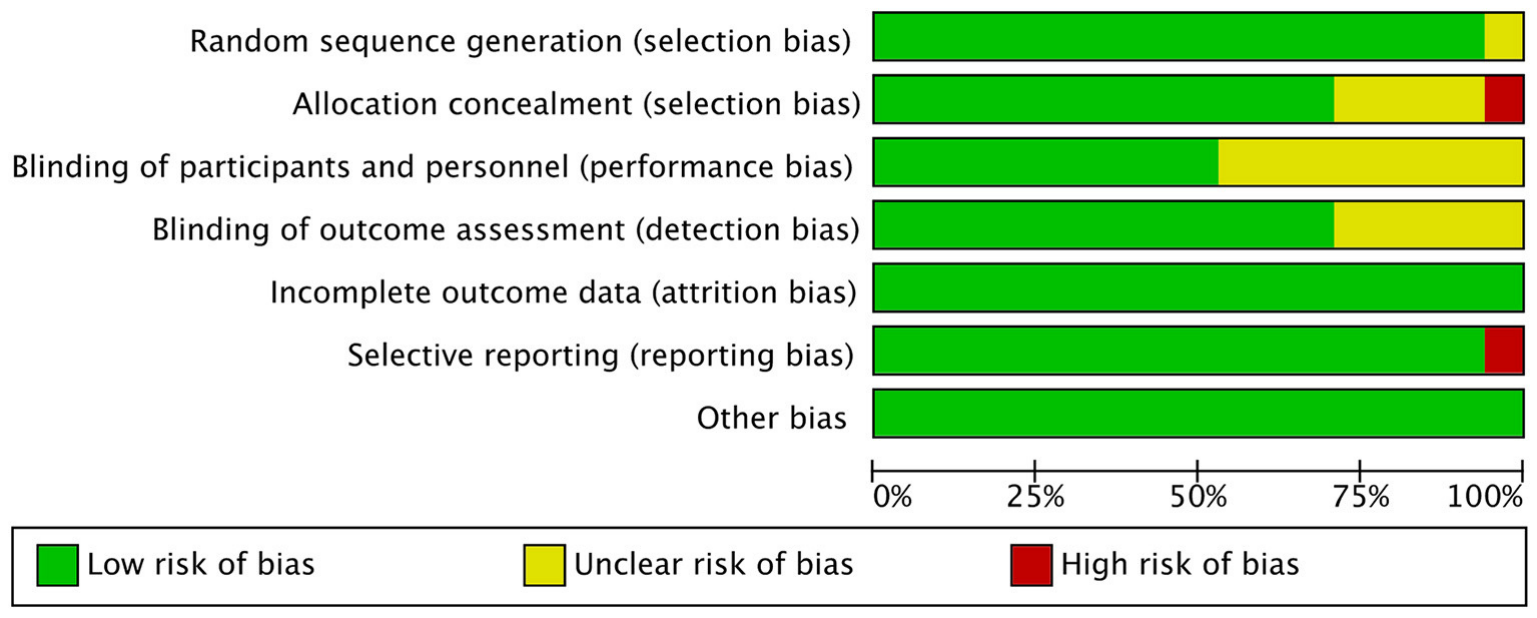
Performance of each type of bias in all studies.

**Figure 3:**
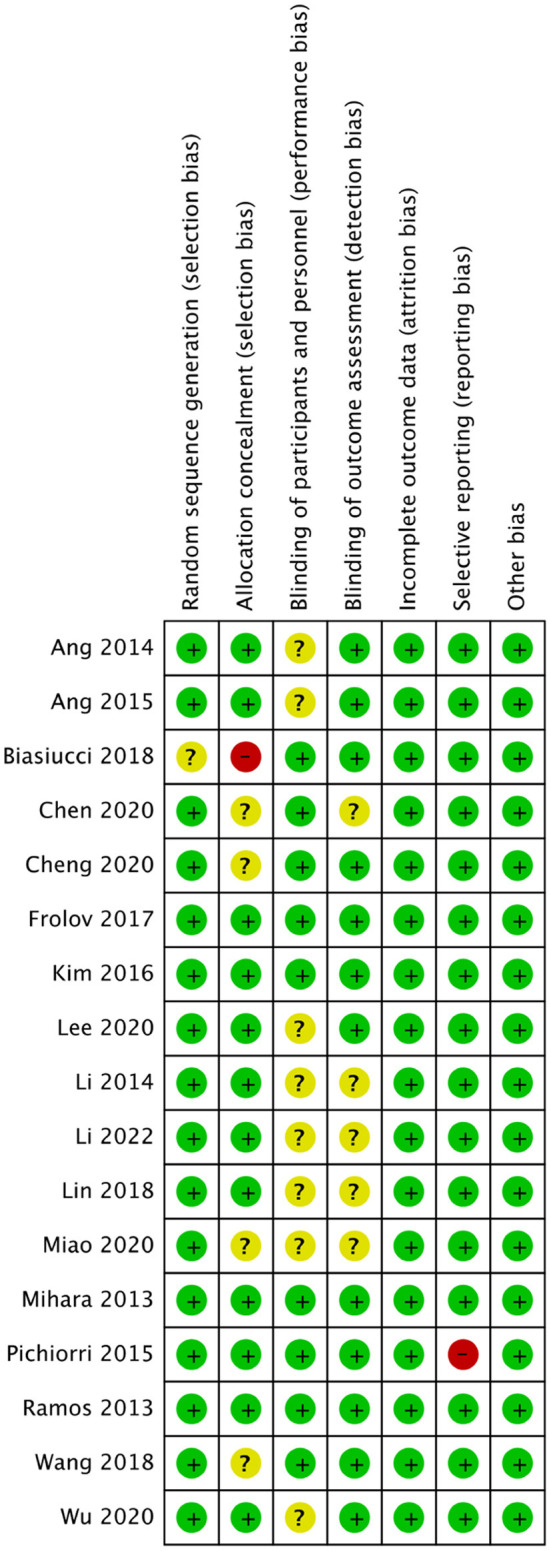
Summary plot of bias in all studies.

The quality assessment of the included RCTs is shown in Figures 2, 3.

### Meta-analysis of treatment effect

The default study event in Revman 5.4.1 software is “adverse event”, and the default forest plot is “Favours experimental” on the left side of the horizontal coordinate and “Favours control” on the right side of the horizontal coordinate.“ However, our study is actually a ”favorable event“, so in the forest plot that appears below, we changed Revman's default to ”Favours control“ on the left side of the horizontal coordinate and ”Favours experimental“ on the right side of the horizontal coordinate.

#### Efficacy of BMI on upper limb motor function

**Figure 4:**
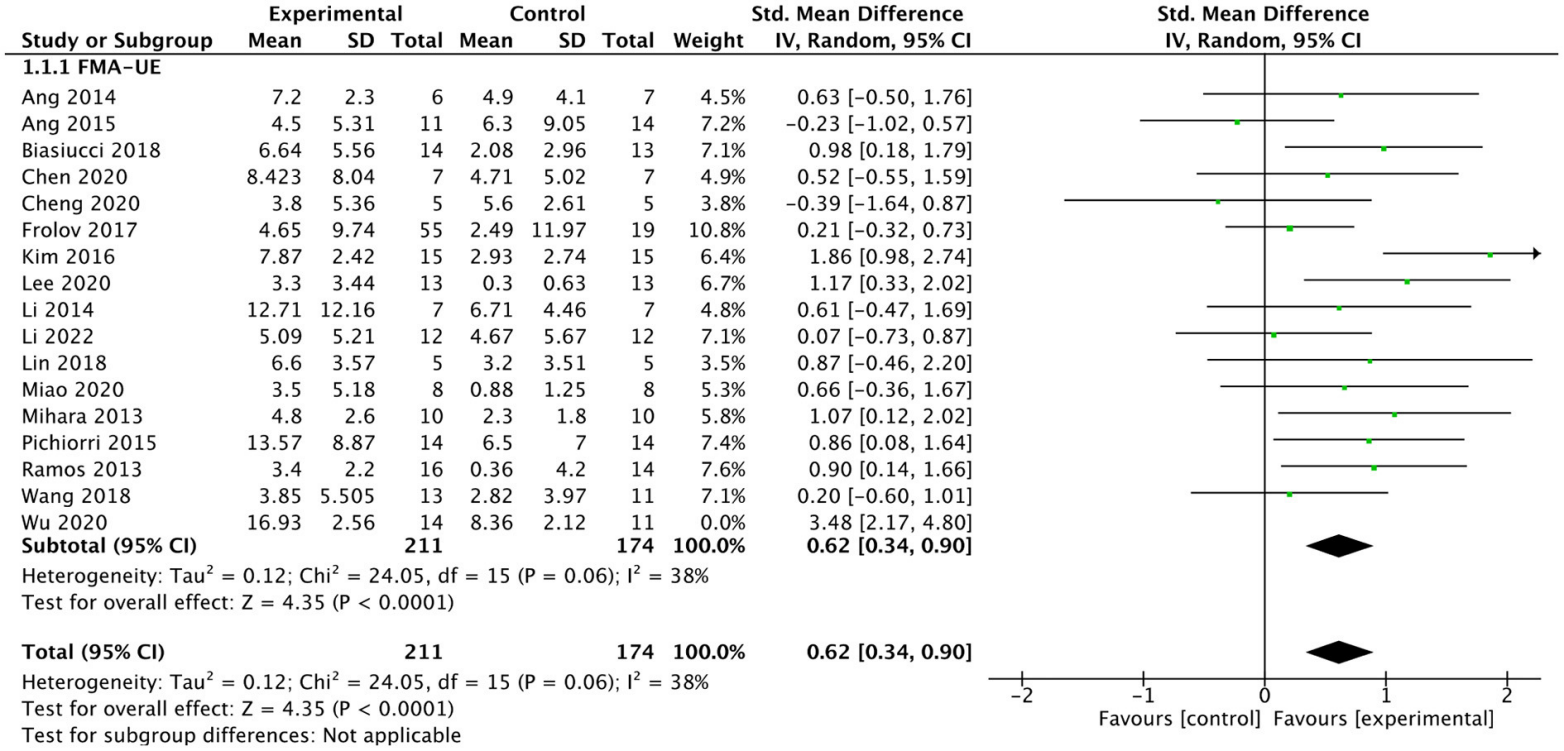
Forest plot for upper lime motor function (Exclude Wu et al.).

**Figure 5:**
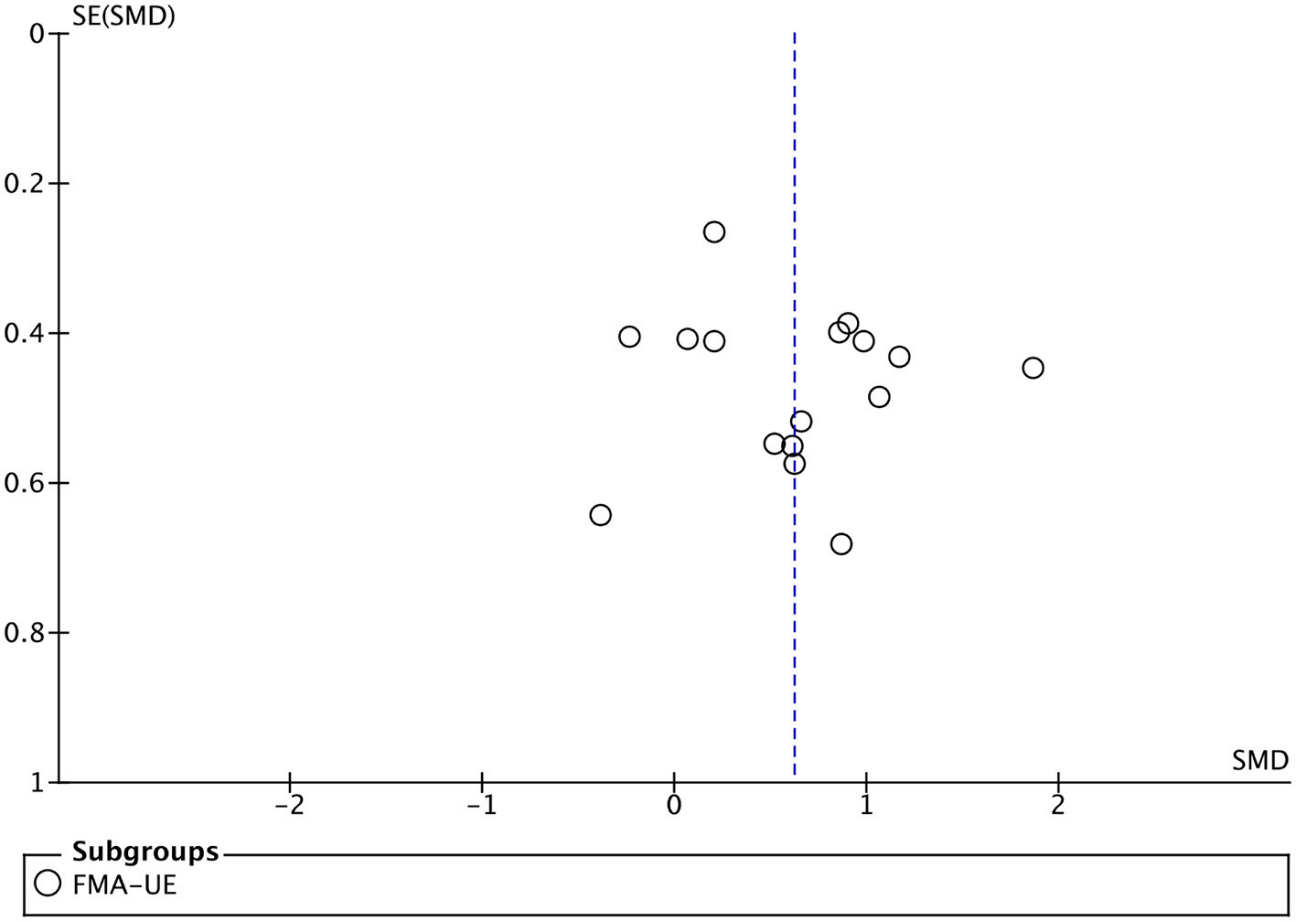
Funnel plot for the publication bias of upper lime motor function.

All included studies used FMA-UE as an outcome measure of upper limb motor function. Seventeen studies involving a total of 410 patients with upper limb motor impairment after stroke evaluated the effect of BMI on FMA-UE. The number of groups that showed improvements above minimal clinically important difference (MCID = 5.25) was eight and five for BMI-based training groups and control groups, respectively. The SMD favors BMI-based training vs. control interventions in 15 out of 17 studies. The combined intervention effect showed that BMI-based training significantly improved upper limb motor function [SMD = 0.75; 95% CI (0.39, 1.10); *I*^2^ = 62%; *p* < 0.0001; random-effects model]. We observed considerable heterogeneity (*I*^2^ = 62%). The sensitivity analysis (removing the single-study method) revealed that the main source of heterogeneity was the study by Wu et al. (2019) with an SMD of 3.48. After excluding this study, the heterogeneity was reduced significantly (*I*^2^ = 38%). The results also showed that BMI-based training significantly improved upper limb motor function [SMD = 0.62, 95% CI (0.34, 0.90); *p* < 0.0001; random-effects mode; Figure 4], and the funnel plot became symmetrical (Figure 5). According to the GRADE, the overall level of evidence for the effect of BMI-based training on upper limb motor function is “Moderate” (Table 2).

**Table 2:** GRADE quality of evidence assessment of individual outcome indicators for the efficacy of BMI-based training in the treatment of upper limb dysfunction.

**Outcome indicator**	**Number of participants**	**Heterogeneity**		**Model of analysis**	**Group effect value**		**Estimated value**	**95% CI**	**Grade**
		***I* ^2^**	***p***		***Z***	***P***			
FMA-UE	410 (17 RCT)	62%	0.004	Random effect	4.13	*p* < 0.0001	0.75 (SMD)	(0.39, 1.10)	Moderate
MBI	80 (3 RCT)	0%	0.81	Random effect	4.61	*p* < 0.00001	1.12 (SMD)	(0.65, 1.60)	Moderate
Adverse effects	126 (4 RCT)	0%	0.73	Fixed effect	0.48	0.63	1.41 (OR)	(0.35, 5.64)	High
Dropout rate	220 (8 RCT)	0%	0.97	Fixed effect	0.41	0.68	1.15 (RR)	(0.59, 2.24)	High

RCT, randomized controlled trials; SMD, standardized mean difference; RR, relative risk; OR, Odds rate; CI, confidence interval; FMA-UE, Fugl-Meyer Assessment-Upper Extremity; MBI, Modified Brathel Index.

#### Subgroup analysis of different post-stroke times

**Figure 6:**
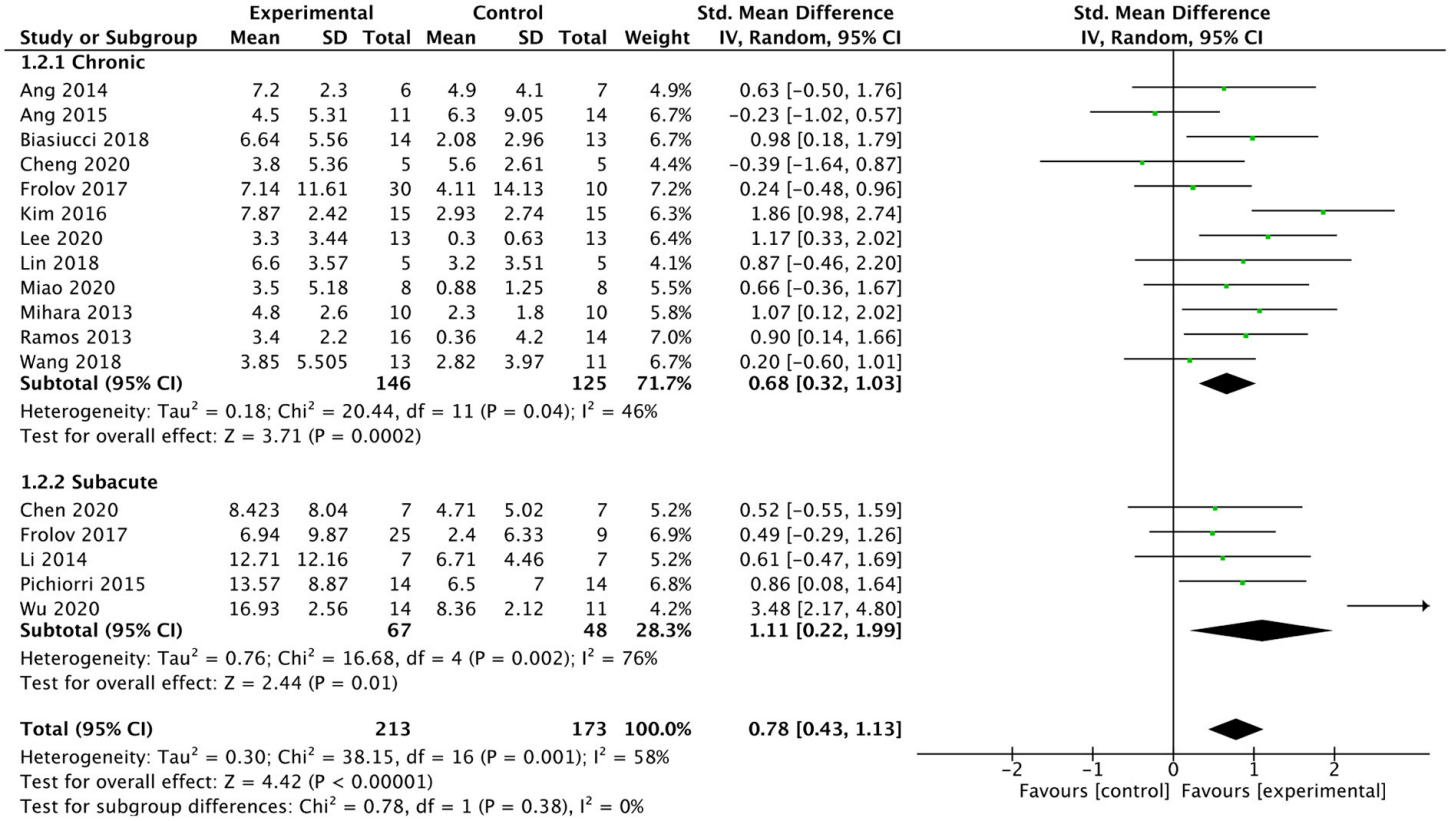
Forest plot for subgroup analysis for upper lime motor function: subacute vs. chronic phase.

We analyzed the effect of BMI-based training on upper extremity function in patients at different post-stroke times (chronic or subacute). Sixteen studies involving a total of 386 patients after stroke were included in the subgroup analysis. For the subgroup analysis (As shown in Figure 6), the results indicated that BMI-based training had a better effect on upper extremity motor function in both chronic [SMD = 0.68; 95% CI (0.32, 1.03), *I*^2^ = 46%; *p* = 0.0002, random-effects model] and subacute [SMD = 1.11; 95%CI (0.22, 1.99); *I*^2^ = 76%; *p* = 0.01; random-effects model] stroke patients compared with control interventions. In subacute stroke patients, BMI-based training showed greater efficacy differences than control interventions, although the difference between the two phases was not significant (*p* = 0.38).

#### Subgroup analysis of different external devices

**Figure 7:**
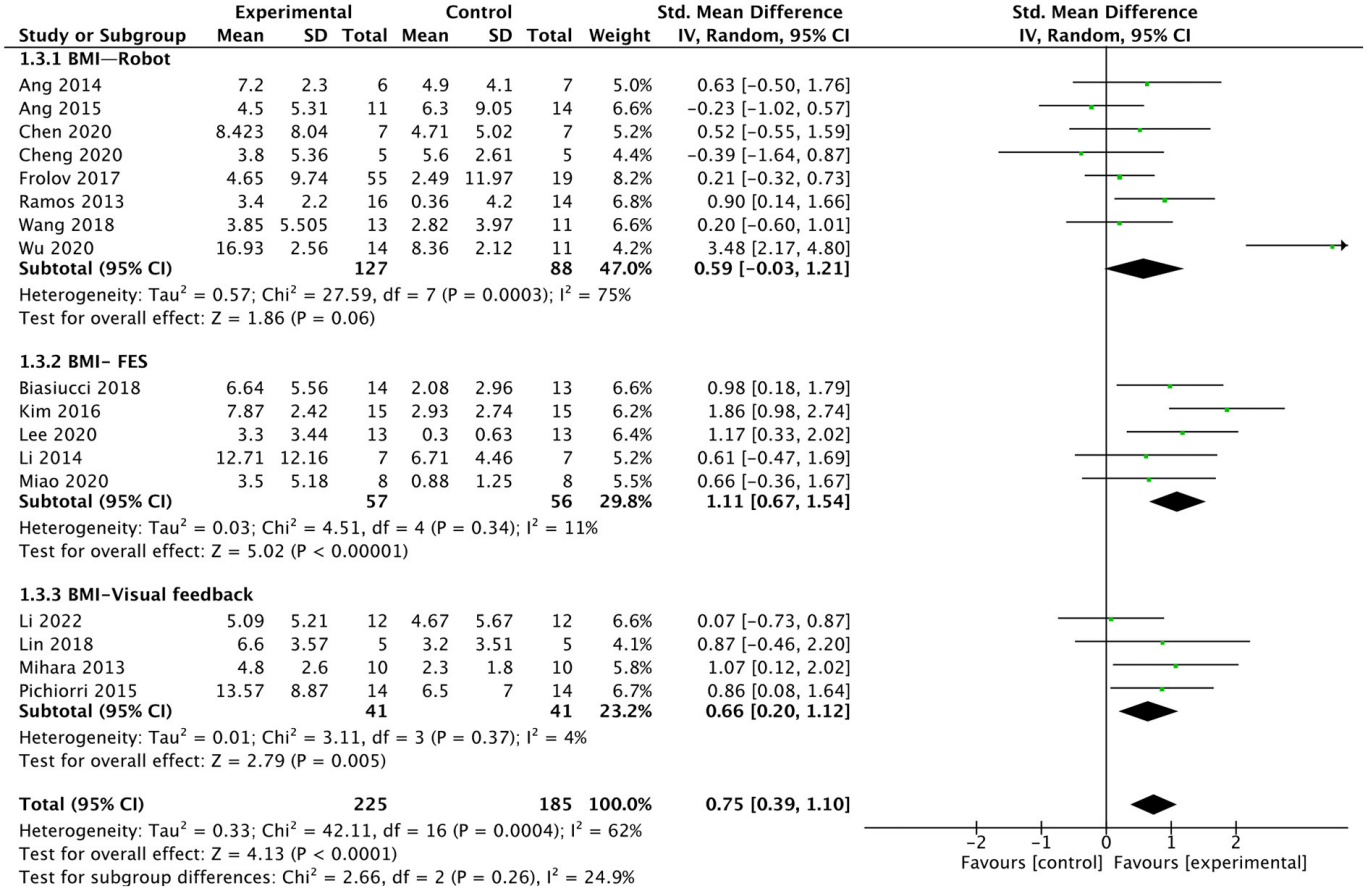
Forest plot for subgroup analysis for upper lime motor function: different external devices: Robot vs. FES (functional electrical stimulation) vs. visual feedback.

We analyzed the effect of BMI combined with the different external devices on upper extremity function in post-stroke patients. We divided external devices into three categories: robot, FES, and visual feedback (As shown in Figure 7), the results indicated that the smallest difference between experimental and control groups can be found for the BMI-robot with an SMD of 0.59, whereas the major difference between the study arms is obtained for BMI-FES subgroup with an SMD of 1.11. Compared with control interventions, BMI combined with FES [SMD = 1.11; 95% CI (0.67, 1.54); *I*^2^ = 11%; *p* < 0.00001; random-effects model]or visual feedback [SMD = 0.66; 95% CI (0.2, 1.12); *I*^2^ = 4%; *p* = 0.005; random-effects model] had a larger effect on up limb motor function recovery. Whereas, BMI-robot did not show greater efficacy in improving upper extremity function compared to control interventions [SMD = 0.59; 95% CI (−0.03, 1.21); *I*^2^ = 75%; *p* = 0.06; random effects model).

### Efficacy of BMI on modified barthel index

**Figure 8:**
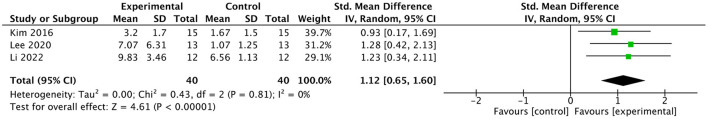
Forest plot for Modified Brathel Index.

Three studies involving a total of 80 post-stroke patients evaluated the effect of BMI-based training on MBI. The results are presented in a forest plot (Figure 8). In two of the three studies, the improvement in MBI scores in the experimental group exceeded the minimal clinically important difference (MCID = 4.26). The combined intervention effect showed that BMI-based training significantly improved ADL [SMD = 1.12; 95% CI (0.65, 1.60); *I*^2^ = 0%; *p* < 0.00001; random-effects model] compared to control interventions. According to the GRADE, the overall level of evidence for the effect of BMI-based training on MBI is “Moderate” (Table 2).

### Meta-analysis of adverse effects

**Figure 9:**
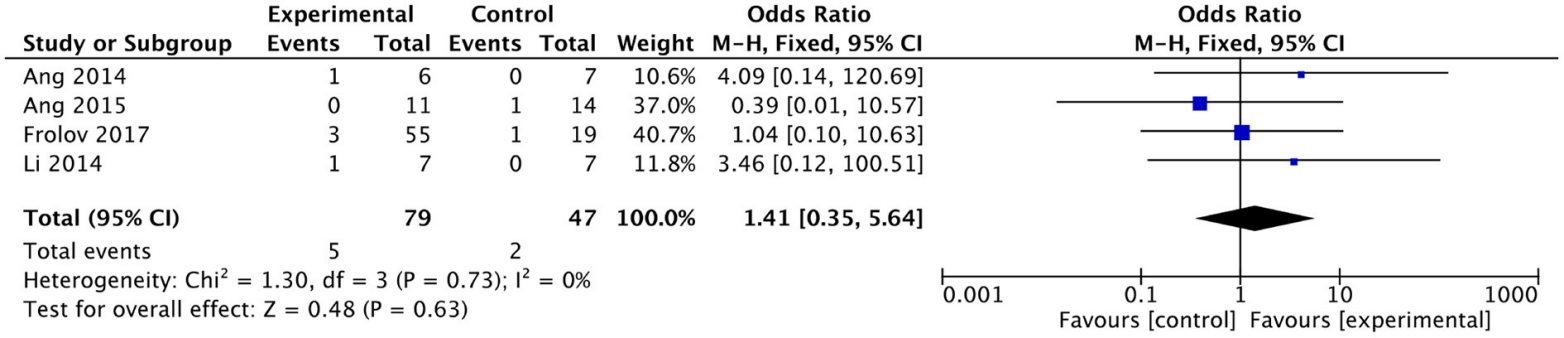
Forest plot for adverse effects.

No serious adverse effects were reported in any of the included studies. Of the remaining four studies, three reported slight discomforts such as mild transient seizures (Ang et al., 2014), headache (Frolov et al., 2017), elevated blood pressure (Frolov et al., 2017), and hypersensitivity to electrode pads (Li et al., 2014) in subjects receiving BMI-based training, and two studies reported slight discomforts such as hemiplegic shoulder pain (Ang et al., 2015) and headache (Frolov et al., 2017) in subjects receiving the control intervention. There was no heterogeneity between studies (*I*^2^ = 0%). The results showed no differences in adverse effects between the BMI-based approaches group and the control group [OR = 1.41; 95% CI (0.35, 5.64); *p* = 0.63; fixed-effects model. Figure 9]. According to the GRADE, the overall level of evidence for the effect of BMI-based training on adverse effects is “High” (Table 2).

### Dropout rate

**Figure 10:**
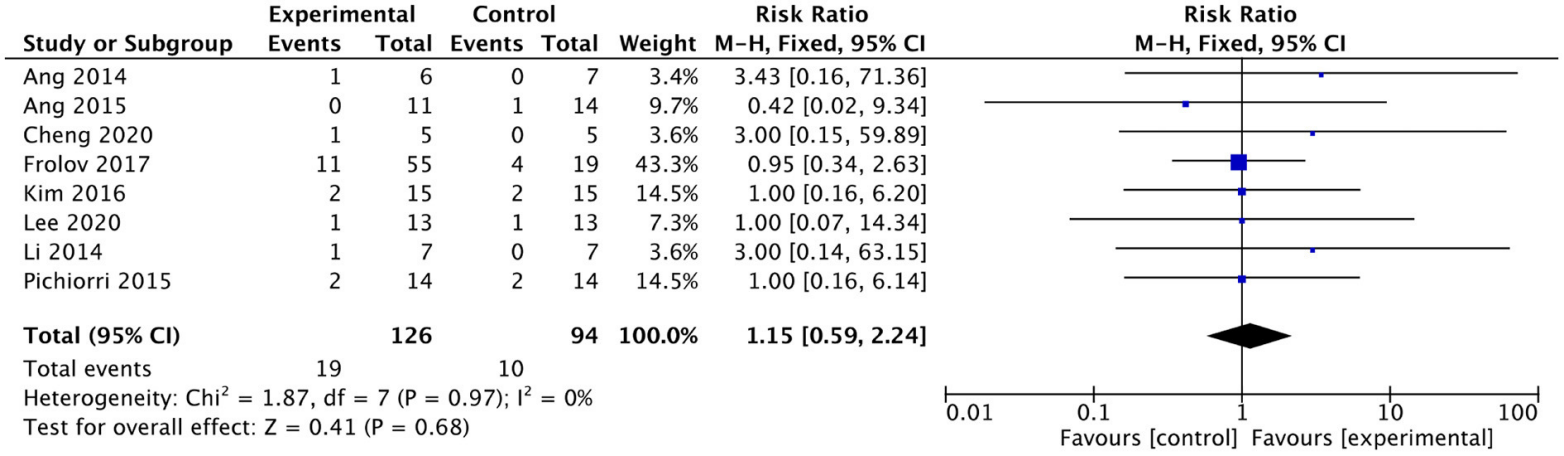
Forest plot for dropout rate.

Eight studies involving a total of 220 patients with post-stroke upper limb dysfunction evaluated the effect of BMI-based approaches on the dropout rate. Heterogeneity of included studies was low (*I*^2^ = 0%), and therefore a fixed-effect model was used for meta-analysis. The results showed no statistically significant difference between the dropout rates of the BMI-based intervention group and the control group [RR = 1.15; 95% CI (0.59, 2.24); *p* = 0.68; fixed-effects model. Figure 10]. According to the GRADE, the overall level of evidence for the effect of BMI-based training on dropout rates is “High” (Table 2).

## Discussion

Various rehabilitation approaches have been proposed due to the heterogeneity of stroke (Szelenberger et al., 2020). Here, we conducted an updated meta-analysis of 17 studies including 410 patients to investigate the efficacy and safety of BMI-based training on upper extremity function in stroke patients. Overall, the results of our meta-analysis showed that BMI-based training significantly promotes recovery of upper extremity motor function (moderate-quality evidence) and ADL (moderate-quality evidence) after stroke. BMI combined with FES or visual feedback may be a better combination for functional recovery than robots. BMI-based training was considered safe and well-tolerated, with no serious adverse effects reported (high-quality evidence).

### Improvement of motor function through BMI-based training

Fugl–Meyer Assessment Scale is a neurological test with good psychometric properties, and a systematic literature review shows that it is the most commonly used measure (Santisteban et al., 2016). Our meta-analysis found that the immediate effect of upper limb motor function (FMA-UE) induced by BMI-based training showed a favorable moderate effect (SMD = 0.75), which was similar to the meta-analysis published in 2018 (SMD = 0.79) and higher than the meta-analysis published in 2020 (SMD = 0.42) (Bai et al., 2020) AND 2022 (SMD = 0.48). Our findings clearly demonstrate that BMI-based training shows greater SMD compared to widely used treatments such as CIMT [SMD = 0.557; 95% CI (0.301, 0.813); *p* < 0.001] (Etoom et al., 2016), mirror therapy [SMD = 0.51; 95% CI (0.29, 0.73); *p* < 0.00001] (Zeng et al., 2018), motor imagery [SMD = 0.36; 95% CI (0.16, 0.55); *p* = 0.0004] (Guerra et al., 2017), supporting its clinical application. Furthermore, it has comparable or significant efficacy compared to other novel rehabilitation techniques such as robot-assisted therapy [SMD = 0.25; 95% CI (0.11, 0.38); *p* = 0.000] (Wu et al., 2021), and virtual reality-based therapy [SMD = 0.42; 95% CI (0.17, 0.67); *p* = 0.00] (Jin et al., 2022). The main source of heterogeneity in this meta-analysis was Wu et al. (2019), which investigated the clinical efficacy of BMI-baesd training and changes in functional brain networks. When this study was removed, the heterogeneity was reduced to 38%, which is within an acceptable range of heterogeneity, and the lower heterogeneity provided a higher quality of evidence. A possible reason for the heterogeneity is Wu et al. included in majority patients with subcortical strokes, which leaves the cortical networks preserved which help the interface between brain and the machine through EEG. Also, the high frequency of the intervention in this study (5 times a week, once a day, for 4 weeks, for a total intervention time of 20 h), while other studies with a total of 20 h of intervention time typically take 6 or 8 weeks to complete (Ang et al., 2014; Li et al., 2014; Cheng et al., 2020).

The first subgroup analysis explored the impact of BMI-based training on upper limb motor function at different stages of stroke. Considering the impact of spontaneous recovery, ”subacute" is defined as within 1–6 months after onset (Cortes et al., 2017), and duration longer than 6 months is considered a chronic phase. Both chronic and subacute strokes have been extensively investigated previously. All studies were included in the subgroup analysis except Li et al. (2022) that did not discuss the efficacy of the subacute and chronic phases separately. The subgroup analyses showed that BMI-based training significantly improved upper limb motor function in both chronic and subacute patients, and demonstrated modest (0.68) and large (1.11) effect sizes, respectively. This suggests that BMI-based training can be used in different stages of stroke, which extends the meaning of BMI in stroke rehabilitation. In chronic and subacute patients, neuroplasticity provides the basis for recovery of motor function after stroke. Plastic reorganization occurs immediately after stroke and provides the basis for recovery of motor function. By providing feedback on the intended movement and thereby restoring the “action-perception coupling”, BMI has already been shown to induce neural plasticity (Ushiba, 2019). Neuroplasticity changes are mainly reflected in changes in functional connectivity and structure of the brain. In the included studies, most of them used EEG and/or MRI to explore the possible neural mechanisms. The mechanisms were divided into the following categories: cortical activation (M1, contralateral cingulate motor areas, supplementary motor areas, premotor cortex, sensorimotor cortex, parietal lobe, frontal lobe, ipsilateral cerebellum) (Mihara et al., 2013; Ramos-Murguialday et al., 2013; Li et al., 2014; Biasiucci et al., 2018; Wang et al., 2018; Chen et al., 2020; Cheng et al., 2020; Lee et al., 2020), enhanced functional connectivity (Biasiucci et al., 2018) (ipsilesional motor areas) (Biasiucci et al., 2018; Wu et al., 2019), and enhanced structural plasticity (corticospinal tract projections, motor pathways) (Biasiucci et al., 2018). A study using near-infrared functional brain imaging also demonstrated that feedback training can alter the excitability of M1 areas (Mihara et al., 2013). In addition, some electrophysiological changes may reflect possible mechanisms of BMI-based training: enhanced SMR desynchronization in sensorimotor cortex and motor areas, enhanced activity in EEG α, β, and μ bands, and movement of event-related potentials toward sensorimotor cortex.

The second subgroup analysis showed that BMI combined with FES or visual feedback was effective in improving hemiplegic upper limb motor function, with FES being more effective. FES is based on the principle that one can artificially compensate for the loss of voluntary motor control by stimulating the paralyzed muscles of the affected limb. The possible mechanism could be explained by the specific role of FES in somatosensory stimulation. Previous studies in healthy individuals support the idea that neural activation in primary sensorimotor areas during motor tasks increases after receiving somatosensory stimuli (Wu et al., 2005). A second possible explanation is that the FES is applied in a bottom-up manner: inducing plasticity in the brain by association with peripheral stimuli. By connecting the FES to the BMI system, muscle contractions become a direct result of the user's intention, turning it into a coupled top-down/bottom-up cycle that can induce brain plasticity more effectively. However, there was no significant difference in the improvement of upper limb motor function between the BMI combined robot and control interventions. The possible reason for this is that there are large differences between studies, for example, although studies have used robots as feedback devices, there are differences between robots, with Ang et al. using the Manus robot, Cheng et al. using the soft robot, and Wu et al. using the exoskeleton. In addition, differences in intervention protocols between studies may also be a reason, with daily intervention time ranging from 0.5 to 1.5 h and total intervention time ranging from 5 to 27 h.

### Improvement of activities of daily living through BMI-based training

MBI is a five-level scale capable of detecting subtle changes in ADL in stroke patients, with excellent test-retest reliability and relatively low random measurement error (Wang et al., 2022; Yang et al., 2022). We reviewed the characteristics of the subjects included in the meta-analysis, who were in the subacute and chronic phases of stroke with moderate-to-severe levels of dysfunction, for which the MBI scale was appropriate. The findings support that BMI-based interventions can be more effective in improving ADL in stroke patients. However, there are still limitations in the number of studies and the small sample size. It is worth noting that the motor tasks used in most studies were only distal upper extremity grasping and rotation, etc. Using ADL as a motor task in the BMI paradigm may be a good therapeutic option for improving ADL ability (Kim et al., 2016; Cheng et al., 2020; Lee et al., 2020). In conclusion, more research is needed to provide evidence for the effects of BMI-based training on ADL.

### Adverse effects and shedding

In terms of treatment safety, all studies supported that the BMI-based intervention was relatively safe, with no serious adverse effects. Only three studies reported minor adverse effects that were reversed after treatment. Fatigue is one of the most common symptoms of post-stroke (Alghamdi et al., 2021). Most of the studies included in the meta-analysis had 30-min or longer interventions, and patients needed to maintain their attention during training, which tended to induce fatigue. Frolov et al. (2017) found that a proportion of subjects reported attention-related fatigue after about 20–30 min of training. Therefore, future studies should consider adding sufficient rest Periods (~15 min after the start of training) to alleviate fatigue and potentially improve the effectiveness of the intervention.

In terms of treatment acceptability, the results of this study suggest that BMI-based training was well-tolerated. In all studies, no one declined the BMI intervention due to dissatisfaction. Of the six studies (Ang et al., 2014; Li et al., 2014; Pichiorri et al., 2015; Kim et al., 2016; Frolov et al., 2017; Lee et al., 2020) that reported dropout events, the reasons can be summarized in the following categories: occurrence of adverse events, early discharge or transfer, and refusal of intervention (personal reasons).

In summary, BMI-based interventions are safe and well-tolerated. (Ang et al., 2014, 2015; Biasiucci et al., 2018; Chen et al., 2020; Cheng et al., 2020).

## Limitations

Several limitations should be considered when interpreting the results of the current study. First, the small sample sizes of most of the included studies may limit the statistical power to detect the effects of BMI-based training on upper extremity function in patients with post-stroke upper extremity dysfunction. Second, fewer studies were available to analyze the effect of BMI-based training on patients performing activities of daily living, and a larger sample and higher quality evidence of BMI-based training is still needed. Third, our paper only performed subgroup analyses of different stages of stroke and combined with external devices, but there was heterogeneity in the results of some subgroup analyses. The efficacy of BMI-based training may also be influenced by other parameters, such as the protocol of the intervention and the severity of the stroke.

## Conclusion

Our analysis showed that BMI-based training improved upper limb motor function and ADL in post-stroke patients, where the combination of BMI and FES or visual feedback showed better efficacy in improving upper limb motor function. Existing studies suggest that the mechanism of BMI is mainly related to activation of the cerebral cortex, improvement of functional connectivity, and structural integrity of the brain. Future studies with larger samples are still needed to provide evidence for the efficacy of BMI-based training in ADL and other areas.

## Data availability statement

The raw data supporting the conclusions of this article will be made available by the authors, without undue reservation.

## Author contributions

Y-lX and Y-xY were responsible for the literature screening, data extraction, statistical analysis, and writing up the article. HJ, L-jG, X-YD, and WQ were responsible for risk of bias assessment. BZ and Y-xW were responsible for planning and guidance on this paper. All authors contributed to the article and approved the submitted version.

## Funding

This work was supported by Research and Development Project of Affiliated Hospital of North Sichuan Medical College (2021ZD014).

## Conflict of interest

The authors declare that the research was conducted in the absence of any commercial or financial relationships that could be construed as a potential conflict of interest.

## Publisher's note

All claims expressed in this article are solely those of the authors and do not necessarily represent those of their affiliated organizations, or those of the publisher, the editors and the reviewers. Any product that may be evaluated in this article, or claim that may be made by its manufacturer, is not guaranteed or endorsed by the publisher.

## References

[B1] Alghamdi I. Ariti C. Williams A. Wood E. Hewitt J. (2021). Prevalence of fatigue after stroke: a systematic review and meta-analysis. Eur. Stroke J. 6, 319–332. 10.1177/23969873211047681 35342803 PMC8948505

[B2] Ang K. K. Chua K. S. Phua K. S. Wang C. Chin Z. Y. Kuah C. W. . (2015). A randomized controlled trial of EEG-based motor imagery brain-computer interface robotic rehabilitation for stroke. Clin. EEG Neurosci. 46, 310–320. 10.1177/1550059414522229 24756025

[B3] Ang K. K. Guan C. Phua K. S. Wang C. Zhou L. Tang K. Y. . (2014). Brain-computer interface-based robotic end effector system for wrist and hand rehabilitation: results of a three-armed randomized controlled trial for chronic stroke. Front. Neuroeng. 7, 30. 10.3389/fneng.2014.00030 25120465 PMC4114185

[B4] Ardern C. L. Buttner F. Andrade R. Weir A. Ashe M. C. Holden S. . (2022). Implementing the 27 PRISMA 2020 Statement items for systematic reviews in the sport and exercise medicine, musculoskeletal rehabilitation and sports science fields: the PERSiST (implementing Prisma in Exercise, Rehabilitation, Sport medicine and SporTs science) guidance. Br. J. Sports Med. 56, 175–195. 10.1136/bjsports-2021-103987 34625401 PMC8862073

[B5] Bai Z. Fong K. N. K. Zhang J. J. Chan J. Ting K. H. (2020). Immediate and long-term effects of BCI-based rehabilitation of the upper extremity after stroke: a systematic review and meta-analysis. J. Neuroeng. Rehabil. 17, 57. 10.1186/s12984-020-00686-2 32334608 PMC7183617

[B6] Bejot Y. Bailly H. Durier J. Giroud M. (2016). Epidemiology of stroke in Europe and trends for the 21st century. Press. Med. 45, e391–e8. 10.1016/j.lpm.2016.10.003 27816343

[B7] Biasiucci A. Leeb R. Iturrate I. Perdikis S. Al-Khodairy A. Corbet T. . (2018). Brain-actuated functional electrical stimulation elicits lasting arm motor recovery after stroke. Nat. Commun. 9, 2421. 10.1038/s41467-018-04673-z 29925890 PMC6010454

[B8] Cervera M. A. Soekadar S. R. Ushiba J. Millan J. D. R. Liu M. Birbaumer N. . (2018). Brain-computer interfaces for post-stroke motor rehabilitation: a meta-analysis. Ann. Clin. Transl. Neurol. 5, 651–663. 10.1002/acn3.544 29761128 PMC5945970

[B9] Chamola V. Vineet A. Nayyar A. Hossain E. (2020). Brain-computer interface-based humanoid control: a review. Sensors. 20, 3620. 10.3390/s20133620 32605077 PMC7374399

[B10] Chaudhary U. Chander B. S. Ohry A. Jaramillo-Gonzalez A. Lule D. Birbaumer N. . (2021). Brain computer interfaces for assisted communication in paralysis and quality of life. Int. J. Neural Syst. 31, 2130003. 10.1142/S0129065721300035 34587854

[B11] Chen S. Cao L. Shu X. Wang H. Ding L. Wang S. H. . (2020). Longitudinal electroencephalography analysis in subacute stroke patients during intervention of brain-computer interface with exoskeleton feedback. Front. Neurosci. 14, 809. 10.3389/fnins.2020.00809 32922254 PMC7457033

[B12] Cheng N. Phua K. S. Lai H. S. Tam P. K. Tang K. Y. Cheng K. K. . (2020). Brain-computer interface-based soft robotic glove rehabilitation for stroke. IEEE Trans. Biomed. Eng. 67, 3339–3351. 10.1109/TBME.2020.2984003 32248089

[B13] Chung E. Lee B. H. Hwang S. (2020). Therapeutic effects of brain-computer interface-controlled functional electrical stimulation training on balance and gait performance for stroke: a pilot randomized controlled trial. Medicine 99, e22612. 10.1097/MD.0000000000022612 33371056 PMC7748200

[B14] Corbett M. S. Higgins J. P. Woolacott N. F. (2014). Assessing baseline imbalance in randomised trials: implications for the Cochrane risk of bias tool. Res. Synth. Methods 5, 79–85. 10.1002/jrsm.1090 26054027

[B15] Corbetta D. Sirtori V. Castellini G. Moja L. Gatti R. (2015). Constraint-induced movement therapy for upper extremities in people with stroke. Cochrane Database Syst Rev. 2017, CD004433. 10.1002/14651858.CD004433.pub3 26446577 PMC6465192

[B16] Cortes J. C. Goldsmith J. Harran M. D. Xu J. Kim N. Schambra H. M. . (2017). A short and distinct time window for recovery of arm motor control early after stroke revealed with a global measure of trajectory kinematics. Neurorehabil. Neural Repair. 31, 552–560. 10.1177/1545968317697034 28506149 PMC5434710

[B17] Daly J. J. Cheng R. Rogers J. Litinas K. Hrovat K. Dohring M. . (2009). Feasibility of a new application of noninvasive Brain Computer Interface (BCI): a case study of train ing for recovery of volitional motor control after stroke. J. Neurol. Phys. Ther. 33, 203–211. 10.1097/NPT.0b013e3181c1fc0b 20208465

[B18] Dawson J. Pierce D. Dixit A. Kimberley T. J. Robertson M. Tarver B. . (2016). Safety, feasibility, and efficacy of vagus nerve stimulation paired with upper-limb rehabilitation after ischemic stroke. Stroke. 47, 143–150. 10.1161/STROKEAHA.115.010477 26645257 PMC4689175

[B19] Ertelt D. Small S. Solodkin A. Dettmers C. McNamara A. Binkofski F. . (2007). Action observation has a positive impact on rehabilitation of motor deficits after stroke. Neuroimage 36(Suppl. 2), T164–T173. 10.1016/j.neuroimage.2007.03.043 17499164

[B20] Etoom M. Hawamdeh M. Hawamdeh Z. Alwardat M. Giordani L. Bacciu S. . (2016). Constraint-induced movement therapy as a rehabilitation intervention for upper extremity in stroke pa tients: systematic review and meta-analysis. Int. J. Rehabil. Res. 39, 197–210. 10.1097/MRR.0000000000000169 27123790

[B21] Feigin V. L. (2008). Stroke: practical management. JAMA 300, 2311. 10.1001/jama.2008.633

[B22] Fleury M. Lioi G. Barillot C. Lecuyer A. A. (2020). Survey on the use of haptic feedback for brain-computer interfaces and neurofeedback. Front. Neurosci. 14, 528. 10.3389/fnins.2020.00528 32655347 PMC7325479

[B23] Frolov A. A. Mokienko O. Lyukmanov R. Biryukova E. Kotov S. Turbina L. . (2017). Post-stroke rehabilitation training with a motor-imagery-based brain-computer interface (BCI)-controlled hand exoskeleton: a randomized controlled multicenter trial. Front. Neurosci. 11, 400. 10.3389/fnins.2017.00400 28775677 PMC5517482

[B24] GBD Stroke Collaborators (2021). Global, regional, and national burden of stroke and its risk factors, 1990-2019: a systematic analysis for the Global Burden of Disease Study 2019. Lancet Neurol. 20, 795–820. 10.1016/S1474-4422(21)00252-0 34487721 PMC8443449

[B25] Gladstone D. J. Danells C. J. Black S. E. (2002). The fugl-meyer assessment of motor recovery after stroke: a critical review of its measurement properties. Neurorehabil. Neural Repair. 16, 232–240. 10.1177/154596802401105171 12234086

[B26] Guerra Z. F. Lucchetti A. L. G. Lucchetti G. (2017). Motor imagery training after stroke: a systematic review and meta-analysis of randomized controlled trials. J. Neurol. Phys. Ther. 41, 205–214. 10.1097/NPT.0000000000000200 28922311

[B27] Guyatt G. H. Oxman A. D. Vist G. E. Kunz R. Falck-Ytter Y. Alonso-Coello P. . (2008). GRADE: an emerging consensus on rating quality of evidence and strength of recommendations. BMJ 336, 924–926. 10.1136/bmj.39489.470347.AD 18436948 PMC2335261

[B28] Higgins J. P. Altman D. G. Gotzsche P. C. Juni P. Moher D. Oxman A. D. . (2011). The Cochrane Collaboration's tool for assessing risk of bias in randomised trials. BMJ 343, d5928. 10.1136/bmj.d5928 22008217 PMC3196245

[B29] Hubner D. Schall A. Prange N. Tangermann M. (2018). Eyes-closed increases the usability of brain-computer interfaces based on auditory event-related potentials. Front. Hum. Neurosci. 12, 391. 10.3389/fnhum.2018.00391 30323749 PMC6172854

[B30] Huedo-Medina T. B. Sánchez-Meca J. Marín-Martínez F. Botella J. (2006). Assessing heterogeneity in meta-analysis: Q statistic or I2 index? Psychol. Methods 11, 193–206. 10.1037/1082-989X.11.2.193 16784338

[B31] Ikbali Afsar S. Mirzayev I. Umit Yemisci O. Cosar Saracgil S. N. (2018). Virtual reality in upper extremity rehabilitation of stroke patients: a randomized controlled trial. J. Stroke Cerebrovasc. Dis. 27, 3473–3478. 10.1016/j.jstrokecerebrovasdis.2018.08.007 30193810

[B32] Jin M. Pei J. Bai Z. Zhang J. He T. Xu X. . (2022). Effects of virtual reality in improving upper extremity function after stroke: a systematic review and meta-analysis of randomized controlled trials. Clin. Rehabil. 36, 573–596. 10.1177/02692155211066534 34898298

[B33] Kang N. Summers J. J. Cauraugh J. H. (2016). Transcranial direct current stimulation facilitates motor learning post-stroke: a systematic review and meta-analysis. J. Neurol. Neurosurg. Psychiatr. 87, 345–355. 10.1136/jnnp-2015-311242 26319437

[B34] Kim T. Kim S. Lee B. (2016). Effects of action observational training plus brain-computer interface-based functional electrical stimulation on paretic arm motor recovery in patient with stroke: a randomized controlled trial. Occup. Ther. Int. 23, 39–47. 10.1002/oti.1403 26301519

[B35] Lee S. H. Kim S. S. Lee B. H. (2020). Action observation training and brain-computer interface controlled functional electrical stimulation enhance upper extremity performance and cortical activation in patients with stroke: a randomized controlled trial. Physiother Theory Pract. (2020) 1–9. 10.1080/09593985.2020.1831114 33026895

[B36] Levin M. F. Sveistrup H. Subramanian S. (2010). Feedback and virtual environments for motor learning and rehabilitation. Schedae 1, 19–36.

[B37] Li C. Wei J. Huang X. Duan Q. Zhang T. (2021). Effects of a brain-computer interface-operated lower limb rehabilitation robot on motor function recovery in patients with stroke. J. Healthc. Eng. 2021, 4710044. 10.1155/2021/4710044 34966524 PMC8712171

[B38] Li M. Liu Y. Wu Y. Liu S. Jia J. Zhang L. . (2014). Neurophysiological substrates of stroke patients with motor imagery-based brain-computer interface training. Int. J. Neurosci. 124, 403–415. 10.3109/00207454.2013.850082 24079396

[B39] Li X. Wang L. Miao S. Yue Z. Tang Z. Su L. . (2022). Sensorimotor rhythm-brain computer interface with audio-cue, motor observation and multisensory feedback for upper-limb stroke rehabilitation: a controlled study. Front. Neurosci. 16, 808830. 10.3389/fnins.2022.808830 35360158 PMC8962957

[B40] Lin B. S. Chen J. L. Hsu H. C. (2018). Novel upper-limb rehabilitation system based on attention technology for post-stroke patients: a preliminary study. IEEE Access 6, 2720–2731. 10.1109/ACCESS.2017.2785122

[B41] Lu J. McFarland D. J. Wolpaw J. R. (2013). Adaptive Laplacian filtering for sensorimotor rhythm-based brain-computer interfaces. J. Neural Eng. 10, 016002. 10.1088/1741-2560/10/1/016002 23220879 PMC3602341

[B42] Lu Y. Xia Y. Wu Y. Pan X. Wang Z. Li Y. . (2022). Repetitive transcranial magnetic stimulation for upper limb motor function and activities of daily living in patients with stroke: a protocol of a systematic review and Bayesian network meta-analysis. BMJ Open 12, e051630. 10.1136/bmjopen-2021-051630 35273041 PMC8915325

[B43] Lyukmanov R. K. Aziatskaya G. A. Mokienko O. A. Varako N. A. Kovyazina M. S. Suponeva N. A. . (2018). Post-stroke rehabilitation training with a brain-computer interface: a clinical and neuropsychological study. Zh. Nevrol. Psikhiatr. Im. S S Korsakova. 118, 43–51. 10.17116/jnevro201811808143 30251977

[B44] Mansour S. Ang K. K. Nair K. P. S. Phua K. S. Arvaneh M. (2022). Efficacy of brain-computer interface and the impact of its design characteristics on poststroke upper-limb rehabilitation: a systematic review and meta-analysis of randomized controlled trials. Clin. EEG Neurosci. 53, 79–90. 10.1177/15500594211009065 33913351 PMC8619716

[B45] McFarland D. J. Wolpaw J. R. (2011). Brain-computer interfaces for communication and control. Commun. ACM 54, 60–66. 10.1145/1941487.1941506 21984822 PMC3188401

[B46] Miao Y. Chen S. Zhang X. Jin J. Xu R. Daly I. . (2020). BCI-based rehabilitation on the stroke in sequela stage. Neural Plast. 2020, 8882764. 10.1155/2020/8882764 33414824 PMC7752268

[B47] Mihara M. Hattori N. Hatakenaka M. Yagura H. Kawano T. Hino T. . (2013). Near-infrared spectroscopy-mediated neurofeedback enhances efficacy of motor imagery-based training in poststroke victims: a pilot study. Stroke 44, 1091–1098. 10.1161/STROKEAHA.111.674507 23404723

[B48] Mutai H. Furukawa T. Nakanishi K. Hanihara T. (2016). Longitudinal functional changes, depression, and health-related quality of life among stroke survivor s living at home after inpatient rehabilitation. Psychogeriatrics 16, 185–190. 10.1111/psyg.12137 26179118

[B49] Nojima I. Sugata H. Takeuchi H. Mima T. (2022). Brain-computer interface training based on brain activity can induce motor recovery in patients with stroke: a meta-analysis. Neurorehabil. Neural Repair 36, 83–96. 10.1177/15459683211062895 34958261

[B50] Pichiorri F. Morone G. Petti M. Toppi J. Pisotta I. Molinari M. . (2015). Brain-computer interface boosts motor imagery practice during stroke recovery. Ann. Neurol. 77, 851–865. 10.1002/ana.24390 25712802

[B51] Platz T. Pinkowski C. van Wijck F. Kim I. H. di Bella P. Johnson G. . (2005). Reliability and validity of arm function assessment with standardized guidelines for the Fugl-Meyer Test, Action Research Arm Test and Box and Block Test: a multicentre study. Clin. Rehabil. 19, 404–411. 10.1191/0269215505cr832oa 15929509

[B52] Purton J. Sim J. Hunter S. M. (2021). The experience of upper-limb dysfunction after stroke: a phenomenological study. Disabil. Rehabil. 43, 3377–3386. 10.1080/09638288.2020.1743775 32223456

[B53] Ramos-Murguialday A. Broetz D. Rea M. Laer L. Yilmaz O. Brasil F. L. . (2013). Brain-machine interface in chronic stroke rehabilitation: a controlled study. Ann. Neurol. 74, 100–108. 10.1002/ana.23879 23494615 PMC3700597

[B54] Santisteban L. Térémetz M. Bleton J. P. Baron J. C. Maier M. A. Lindberg P. G. . (2016). Upper limb outcome measures used in stroke rehabilitation studies: a systematic literature review. PLoS ONE 11, e0154792. 10.1371/journal.pone.0154792 27152853 PMC4859525

[B55] Szelenberger R. Kostka J. Saluk-Bijak J. Miller E. (2020). Pharmacological interventions and rehabilitation approach for enhancing brain self-repair and stroke recovery. Curr. Neuropharmacol. 18, 51–64. 10.2174/1570159X17666190726104139 31362657 PMC7327936

[B56] Tani M. Ono Y. Matsubara M. Ohmatsu S. Yukawa Y. Kohno M. . (2018). Action observation facilitates motor cortical activity in patients with stroke and hemiplegia. Neurosci. Res. 133, 7–14. 10.1016/j.neures.2017.10.002 29031830

[B57] Thieme H. Morkisch N. Mehrholz J. Pohl M. Behrens J. Borgetto B. . (2018). Mirror therapy for improving motor function after stroke. Cochrane Database Syst Rev. 7, CD008449. 10.1002/14651858.CD008449.pub3 29993119 PMC6513639

[B58] Ushiba J. (2019). Brain-machine interface and neuro-rehabilitation. Brain Nerve. 71, 793–804. 10.11477/mf.1416201352 31289253

[B59] van Dokkum L. E. H. Ward T. Laffont I. (2015). Brain computer interfaces for neurorehabilitation - its current status as a rehabilitation strategy post-stroke. Ann. Phys. Rehabil. Med. 58, 3–8. 10.1016/j.rehab.2014.09.016 25614021

[B60] Verbaarschot C. Tump D. Lutu A. Borhanazad M. Thielen J. van den Broek P. . (2021). A visual brain-computer interface as communication aid for patients with amyotrophic lateral sclerosis. Clin. Neurophysiol. 132, 2404–2415. 10.1016/j.clinph.2021.07.012 34454267

[B61] Vilela M. Hochberg L. R. (2020). Applications of brain-computer interfaces to the control of robotic and prosthetic arms. Handb. Clin. Neurol. 168, 87–99. 10.1016/B978-0-444-63934-9.00008-1 32164870

[B62] Wang X. Wong W. W. Sun R. Chu W. C. Tong K. Y. (2018). Differentiated effects of robot hand training with and without neural guidance on neuroplasticity patterns in chronic stroke. Front. Neurol. 9, 810. 10.3389/fneur.2018.00810 30349505 PMC6186842

[B63] Wang Y. C. Chang P. F. Chen Y. M. Lee Y. C. Huang S. L. Chen M. H. . (2022). Comparison of responsiveness of the Barthel Index and modified Barthel Index in patients with stroke. Disabil. Rehabil. (2022) 1–6. 10.1080/09638288.2022.2055166 35357990

[B64] Winstein C. J. Stein J. Arena R. Bates B. Cherney L. R. Cramer S. C. . (2016). Guidelines for adult stroke rehabilitation and recovery: a guideline for healthcare professionals from the American Heart Association/American Stroke Association. Stroke 47, e98–e169. 10.1161/STR.0000000000000098 27145936

[B65] Wu C. W. van Gelderen P. Hanakawa T. Yaseen Z. Cohen L. G. (2005). Enduring representational plasticity after somatosensory stimulation. Neuroimage 27, 872–884. 10.1016/j.neuroimage.2005.05.055 16084740

[B66] Wu J. Cheng H. Zhang J. Yang S. Cai S. (2021). Robot-assisted therapy for upper extremity motor impairment after stroke: a systematic review and meta-analysis. Phys. Ther. 101, pzab010. 10.1093/ptj/pzab010 33454787

[B67] Wu Q. Yue Z. Ge Y. Ma D. Yin H. Zhao H. . (2019). Brain functional networks study of subacute stroke patients with upper limb dysfunction after comprehensive rehabilitation including BCI training. Front. Neurol. 10, 1419. 10.3389/fneur.2019.01419 32082238 PMC7000923

[B68] Yang C. M. Wang Y. C. Lee C. H. Chen M. H. Hsieh C. L. A. (2022). comparison of test-retest reliability and random measurement error of the Barthel Index and modified Barthel Index in patients with chronic stroke. Disabil. Rehabil. 44, 2099–2103. 10.1080/09638288.2020.1814429 32903114

[B69] Yang H. Chen Y. Wang J. Wei H. Chen Y. Jin J. . (2021). Activities of daily living measurement after ischemic stroke: Rasch analysis of the modified Barthel Index. Medicine 100, e24926. 10.1097/MD.0000000000024926 33655956 PMC7939171

[B70] Yang W. Zhang X. Li Z. Zhang Q. Xue C. Huai Y. . (2021a). The effect of brain-computer interface training on rehabilitation of upper limb dysfunction after stroke: a meta-analysis of randomized controlled trials. Front. Neurosci. 15, 766879. 10.3389/fnins.2021.766879 35197817 PMC8859107

[B71] Zeng W. Guo Y. Wu G. Liu X. Fang Q. (2018). Mirror therapy for motor function of the upper extremity in patients with stroke: a meta-analysis. J. Rehabil. Med. 50, 8–15. 10.2340/16501977-2287 29077129

